# Rapid neurological recovery in Guillain-Barré syndrome treated with efgartigimod

**DOI:** 10.1038/s41598-026-44163-7

**Published:** 2026-03-19

**Authors:** Yifan Cheng, Wenyu Li, Siyu Xie, Shunyuan Guo, Chunrong Li, Manman Zhang, Yiqi Wang

**Affiliations:** 1 Center for Rehabilitation Medicine, Department of Neurology, Zhejiang Provincial People’s hospital (Affiliated People’s Hospital, Hangzhou Medical College), No.159 Shangtang Road, Hangzhou, China; 2 Department of Neurology, Zhejiang University School of Medicine Sir Run Run Shaw Hospital, No.3 East Qingchun Road, Hangzhou, China; 3 The Second Clinical Medical College of Hangzhou Normal University, No.2318 Yuhangtang Road, Hangzhou, China

**Keywords:** Guillain–Barré syndrome, Efgartigimod, Intravenous immunoglobulin, Plasma exchange, Neuroimmunology, Peripheral nervous system

## Abstract

Efgartigimod, a neonatal Fc receptor inhibitor that accelerates IgG degradation, remains understudied in treatment of Guillain-Barré Syndrome (GBS). This study aimed to evaluate the efficacy and safety of efgartigimod in GBS patients. A retrospective analysis included 17 GBS patients (5 patients treated by efgartigimod, 8 patients treated by IVIg, 4 patients treated by PE). Primary outcome is time to a one-point improvement in GBS-DS and time to regain unaided walking ability. GBS-DS score, Inflammatory Neuropathy Cause and Treatment (INCAT) disability score, Medical Research Council (MRC) scores, the proportions of patients with INCAT score ≤ 2 and those achieving full MRC score were recorded at baseline and at 1 week, 1 month, and 3 months post-treatment. Adverse reactions were monitored throughout the treatment period. The patients treated by efgartigimod demonstrated significantly faster improvement of 1-point on the GBS-DS (4.00 days vs. IVIg: 7.00 days, PE: 11.50 days; *p* = 0.033) and higher proportions of patients with INCAT scores ≤ 2 (80% vs. 12.5% [IVIg], 25% [PE]; *p* = 0.039) and full MRC scores (80% vs. 12.5% [IVIg]; *p* = 0.045) at 1 week. Adverse events were mild (20% vs.50% PE). Efgartigimod offers rapid symptom relief and functional recovery in GBS compared to IVIg and PE. These findings highlight its potential as a novel therapeutic option.

**Supplementary Information:**

The online version contains supplementary material available at 10.1038/s41598-026-44163-7.

## Introduction

Guillain-Barré syndrome (GBS) is an acute autoimmune disorder of the peripheral nervous system, which is triggered by infections and vaccinations and is characterized by rapid-onset flaccid tetraplegia^1,2^.The global yearly incidence of GBS ranges from 0.44^3^ to 3.25^4^ cases per 100,000 individuals, resulting in approximately 100,000 new cases worldwide annually^5^.

Intravenous immunoglobulin (IVIg) and plasma exchange (PE) are considered to be the only two proven therapies for GBS^6^. PE is a blood separation technique, it takes at least 2 to 4 exchanges to become effective^7^. A multicenter controlled trail in 1980s showed that PE can rapidly improve motor function and reduce the need for prolonged mechanical ventilation^8^. IVIg is purified from human plasma pooled from at least 1000 healthy donors^6^ and has also been shown to be effective in treating GBS^9^. However, these therapies exhibit certain limitations. IVIg can’t avoid continued disease progression in time in some cases^10,11^ and about 20% of patients are left with substantial disability after either treatment^8,9^.Treatment associated side effect, such as arrhythmias, hypotension, thromboembolic events and renal failure often clinically observed^12–14^. In addition, PE is limited to special equipment and plasma supplies. IVIg is also in limited supply. Exploration of alternative treatments that can halt rapid disease progression and improve GBS patient’s outcomes is necessary.

Efgartigimod is a high-affinity recombinant human IgG1 Fc fragment that inhibits the neonatal Fc receptor (FcRn)^15^. It contains engineered amino acid substitutions in the Fc region (MST-HN = YTE-KF), resulting in increased affinity for FcRn and enhanced IgG clearance^16^. Several autoimmune disorders have been successfully treated with efgartigimod, including generalized myasthenia gravis (MG)^17^, pemphigus vulgaris and pemphigus foliaceus^18^, immune thrombocytopenia^19^, and chronic inflammatory demyelinating polyradiculoneuropathy (CIDP)^20^.Researchers have investigated the ability of efgartigimod to lower autoantibody levels and enhance neurological recovery, positioning it as a novel therapeutic approach for managing GBS. Recently several case reports show that efgartigimod demonstrated significant neurological relief in the treatment of GBS^21,22^. However, it remains unclear whether efgartigimod is actually more effective in treating GBS than traditional treatments including IVIg and PE.

Therefore, our study was conducted to evaluate the efficacy and safety of efgartigimod in patients with GBS compared with traditional treatments.

## Materials and methods

### Study design

Retrospective continuous data were collected for patients aged > 18 years diagnosed with GBS at Zhejiang Provincial People’s Hospital and Run Run Shaw Hospital, Zhejiang University School of Medicine, and within 4 weeks of symptom onset between November 2022 and August 2024. The diagnosis and clinical classification of GBS followed diagnostic criteria outlined in the 2023 European Academy of Neurology/Peripheral Nerve Society guidelines^23^.

In this study, we used the GBS-Disability Scale(GBS-DS) described by Hughes et al., defined as follows: 0, healthy; 1, minor symptoms or signs but able to run; 2, able to walk 10 m independently; 3, able to walk 10 m with a walker or support; 4, bed chair-bound; 5, requiring assisted respiration; and 6, deceased^24^. All patients had GBS-DS score of 3 or higher at presentation.

Exclusion criteria included evidence of other peripheral neuropathies, associated malignancies, or congenital complement defects. Patients who received both PE and IVIg and who didn’t receive any immunotherapy were excluded.

### Treatment

All GBS patients initiate immunotherapy within three days after confirmation of diagnosis. The immunotherapy regimen included IVIg, PE and intravenous infusion of efgartigimod. The treatment regimen was determined based on the patient’s condition, financial burden, and patient’s acceptance of invasive procedures. The patient made the final immunotherapy regimen and signs the informed consent form.

We divided GBS patients into the following three groups according to the different immunotherapy regimen:

IVIg group: patients received IVIg at a dose of 0.4 g/kg per day for five days. PE group: patients underwent PE of 50 mL/kg once every other day lasting for 3–5 times. Efgartigimod group (hereinafter referred to as the EFG group), patients received 15 mg/kg of efgartigimod on day 1 and day 5. Those patients treated with PE follow by efgartigimod were classified into EFG group. In those patients, the day of efgartigimod treatment was considered the initiation of immunotherapy.

### Data collection

#### Baseline clinical data

Baseline clinical data collected included gender, age at onset, clinical classification of GBS (GBS, Miller Fisher syndrome [MFS], and GBS/MFS overlap), core symptoms (limb weakness or ataxia), other concomitant symptoms (facial paralysis, pain, limb numbness, dysphagia, dysarthria, dyspnea), presence of serum ganglionic anhydride antibodies, albumin-cytologic dissociation, and the time from symptom onset to immunotherapy initiation.

#### Efficacy outcomes

Primary outcome is time to a one-point improvement in GBS-DS and time to recover unaided walking. “Unaided walking” was defined as independent walking without scheduled assessments at least for 10 m, which corresponded to GBS-DS level 2. GBS-DS score, Inflammatory Neuropathy Cause and Treatment (INCAT) disability score^25^, and Medical Research Council (MRC) scores^26^ were recorded at baseline (prior to immunotherapy) and at 1 week, 1 month, and 3 months post-treatment. The proportions of patients with INCAT score ≤ 2 and those achieving full MRC score were recorded at baseline and at 1 week, 1 month, and 3 months after immunotherapy.

#### Safety outcomes

All patients were monitored for adverse reactions throughout the treatment period, and specific adverse events were documented.

### Statistical analysis

Clinical baseline characteristics of the IVIg, PE, and EFG groups were assessed for statistical differences. Time to one-point improvement in GBS-DS, time to recover unaided walking and GBS-DS, INCAT, and MRC scores at baseline, 1 week,1 month, and 3 months post treatment, were compared among the three groups. The proportions of patients with INCAT score ≤ 2, and those with MRC reaching full scores were also compared. Time to other symptom disappeared and adverse reaction rates were compared among the groups.

Categorical data were described as frequency (N) and percentages (%), and were compared using the chi-square test or Fisher’s exact test when appropriate. The normality of continuous data was assessed by the Shapiro-Wilk test. Normally distributed continuous data were described as mean ± standard deviation (SD), while non-normally distributed data were described as median and interquartile range (Median [IQR]). ANOVA was used for comparing of normally distributed continuous data among groups, while the Kruskal-Walli’s test was used for non-normally distributed data. Bonferroni correction was applied for pairwise comparisons. All tests were two-sided with a significance level of α = 0.05. Statistical analyses were performed using R (Version 4.4.0).

The study protocol was approved by the ethics committees of all participating centers, and informed consent was obtained from all patients. All methods were performed in accordance with the relevant guidelines and regulations.

## Results

**Fig. 1:**
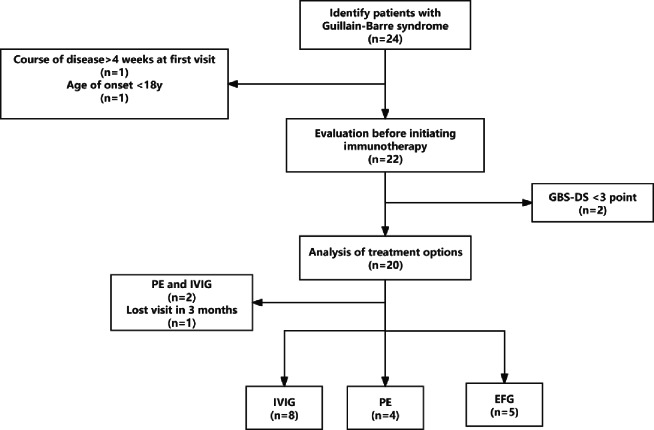
Study flowchart.

This retrospective study included 24 GBS patients who were admitted between November 2022 and August 2024. 1 patient with a disease duration exceeding four weeks at the first visit and one case under 18 years old were excluded. Of the remaining 22 patients, 2 patients with GBS-DS scores < 3 were also excluded. Among the remaining 20 patients, 2 patients who received PE followed by IVIg were excluded, along with one case lost to follow-up within three months. Finally, 17 patients were included in the analysis, with 8 in the IVIg group, 4 in the PE group, and 5 in the EFG group. (Fig. 1).

The clinical baseline characteristics of the 17 patients were as follows: 7 males and 10 females, with ages ranging from 26 to 85 years. There were 14 GBS cases, 1 MFS case, and 2 cases of GBS/MFS overlap. Core symptoms included limb weakness in 14 patients and ataxia in 3. Other symptoms included pain in 3 patients, facial paralysis in 2, diplopia and ptosis in 1, dysphagia and dysarthria in 4, dyspnea in 2, and urinary retention in two 2.

Serum ganglioside antibody testing was positive in 6 patients, with the detected antibodies including anti-GQ1b IgG, anti-GM1 IgG, anti-GM3 IgG, anti-GM4 IgG, anti-GT1a IgG, and anti-GD1b IgG. Albumin-cytologic dissociation was present in 12 patients, and the time from symptom onset to initiation of immunotherapy ranged from 4 to 27 days. The clinical information of patients in EFG group were specially listed in table S1. Among the 5 patients in EFG group, 3 patients (patient 1, patient 2 and patient 3) were treated with efgartigimod monotherapy, and 2 patients (patient 4 and patient 5) received PE treatment followed by efgartigimod treatment. (Table S1). No significant differences in baseline characteristics were observed among IVIg, PE and EFG groups (Table 1).

**Table 1:** Clinical baseline of EFG group, PE group and IVIg group.

Variables	EFG(*n* = 5)	PE(*n* = 4)	IVIg(*n* = 8)	Statistic	*P*
Age	50.80 ± 19.69	58.75 ± 16.17	60.88 ± 16.93	F = 0.52	0.606
INCAT score, Mean ± SD	6.80 ± 2.59	8.25 ± 1.50	7.12 ± 1.89	F = 0.61	0.556
INCAT2, n (%)	0(0%)	0(0%)	0(0%)	-	1.000
MRC, M (Q_1_, Q_3_)	50.0(36.0,60.0)	49.0(39.0,51.5)	43.0(42.0,48.0)	X^2^=0.46#	0.793
MRC60, n (%)	2(40%)	0(0%)	0(0%)	-	0.067
Treatment days, M (Q_1_, Q_3_)	8.0(4.0,16.0)	7.0(5.0,9.8)	5.5(4.8,7.0)	X^2^=0.46#	0.794
GBS-DS, M (Q_1_, Q_3_)	4.0(4.0,4.0)	4.0(3.8,4.0)	4.0(3.0,4.0)	X^2^=0.52#	0.77
Male, n (%)	3(60.0%)	3(75.0%)	4(50.0%)	-	0.827
Symptom, n (%)				-	0.394
limb weakness	3(60.0%)	4(100.0%)	7(87.5%)		
ataxia	2(40.0%)	0(0.0%)	1(12.5%)		
GBS subtype, n (%)				-	0.671
GBS	3(60.0%)	4(100.0%)	7(87.5%)		
MFS	1(20.0%)	0(0.0%)	0(0.0%)		
GBS//MFS	1(20.0%)	0(0.0%)	1(12.5%)		
Albumin cytologic dissociation, n (%)	4(80.0%)	3(75.0%)	5(62.5%)	-	1.000
Antibody positive, n (%)	2(40.0%)	2(50.0%)	2(25.0%)	-	0.819

F: ANOVA, #: Kruskal-waills test, -: Fisher exact.

SD: standard deviation, M: Median, Q_1_: 1st Quartile, Q_3_: 3st Quartile *p < 0.05, **p < 0.01.

EFG: Efgartigimod; PE: Plasma Exchanges; IVIg: Intravenous immunoglobulin.

GBS: Guillain-Barre syndrome; MFS: Miller Fisher syndrome; GBS/MFS: GBS/MFS overlap; INCAT: Inflammatory Neuropathy Cause and Treatment; GBS-DS: GBS disability grade score; MRC: Medical Research Council scores.

### Efficacy assessment

The time from immunotherapy initiation to a one-point improvement in GBS-DS score differed significantly among the three groups (EFG group: 4.00 [3.00, 5.00] days, IVIg group: 7.00 [6.50, 19.50] days PE group: 11.50 [6.25, 64.50] days, *p* = 0.033). The EFG group recovered unaided walking earlier than the IVIg and PE group, although the difference was not statistically significant (*p* = 0.23).

At the first week post-treatment, the proportion of patients with INCAT scores ≤ 2 varied significantly among the three groups (EFG group: 80% vs. PE group: 25% vs. IVIg group: 12.5%, *p* = 0.039). The proportion of patients achieving full MRC score within one week also showed a significant trend among the groups (EFG group, 80%; PE group, 50%; IVIg group,12.5%, *p* = 0.045). the proportion of unaided walking patients in EFG group was higher than PE and IVIg group without statistically differences (80% vs. 50% vs. 37.5%, *p* = 0.39). the GBS-DS and INCAT scores in EFG group were slightly lower than PE and IVIg group. The MRC scores in EFG group were slightly higher than PE and IVIg group. At 1 month and 3-month post-treatment, there were no significant differences in all effectiveness indicators among the three groups. (Table 2).

**Table 2:** Outcome measure of EFG group, PE group and IVIg group.

	Variables	EFG(*n* = 5)	PE(*n* = 4)	IVIg(*n* = 8)	Statistic	*P*
	Time to 1point improvement in GBS-DS (days)	4.00 (3.00,5.00)	11.50 (6.25,64.50)	7.00 (6.50,19.50)	X^2^=6.81#	0.033*
	Time to recover unaided walking(days)	6.00 (5.00,7.00)	21.00 (6.75,101.25)	19.00 (7.00,97.50)	X^2^=2.92#	0.230
1 week	GBS-DS	2.00(2.00,2.00)	3.00(2.00,4.00)	3.00(2.00,4.00)	X^2^=1.68#	0.430
	INCAT	2.00 ± 3.94	6.00 ± 4.08	4.75 ± 2.05	F = 1.96	0.180
	MRC	55.60 ± 9.84	46.50 ± 23.17	51.88 ± 5.41	F = 0.59	0.570
	INCAT2, n (%)	4(80.0%)	1(25.0%)	1(12.5%)	-	0.039*
	MRC60, n (%)	4(80.0%)	2(50.0%)	1(12.5%)	-	0.045*
	Unaided walking, n (%)	4(80.0%)	2(50.0%)	3(37.5%)	-	0.390
1 month	GBS-DS	0.00(0.00,0.00)	1.00(0.00,3.50)	0.50(0.00,3.00)		0.660
	INCAT	1.00 ± 2.24	3.00 ± 4.76	2.38 ± 2.45	F = 0.53	0.600
	MRC	57.20 ± 6.26	47.50 ± 23.69	55.88 ± 5.46	F = 0.84	0.450
	INCAT2, n (%)	4(80.0%)	3(75.0%)	4(50.0%)	-	0.540
	MRC60, n (%)	4(80.0%)	2(50.0%)	4(50.0%)	-	0.570
	Unaided walking, n (%)	4(80.0%)	3(75.0%)	5(62.5%)	-	1.000
3 months	GBS-DS	0.00(0.00,0.00)	0.50(0.00,1.75)	0.00(0.00,2.25)	X^2^=0.98#	0.610
	INCAT	0.60 ± 1.34	2.75 ± 4.86	2.00 ± 2.33	F = 0.67#	0.530
	MRC	58.40 ± 3.58	48.00 ± 24.00	56.25 ± 5.18	F = 0.95	0.410
	INCAT2, n (%)	4(80.0%)	3(75.0%)	5(62.5%)	-	1.000
	MRC60, n (%)	4(80.0%)	3(75.0%)	5(62.5%)	-	1.000
	Unaided walking, n (%)	5(100%)	3(75.0%)	6(75.0%)	-	0.560

F: ANOVA; #: Kruskal–Wallis test; -: Fisher’s exact test. *p < 0.05, **p < 0.01.

GBS-DS: Guillain-Barre syndrome disability grade score; INCAT Inflammatory Neuropathy Cause and Treatment; MRC: Medical Research Council (MRC)scores; INCAT2: proportion of patients with INCAT scores ≤ 2; MRC60: proportion of patients with MRC reaching full marks.

**Fig. 2:**
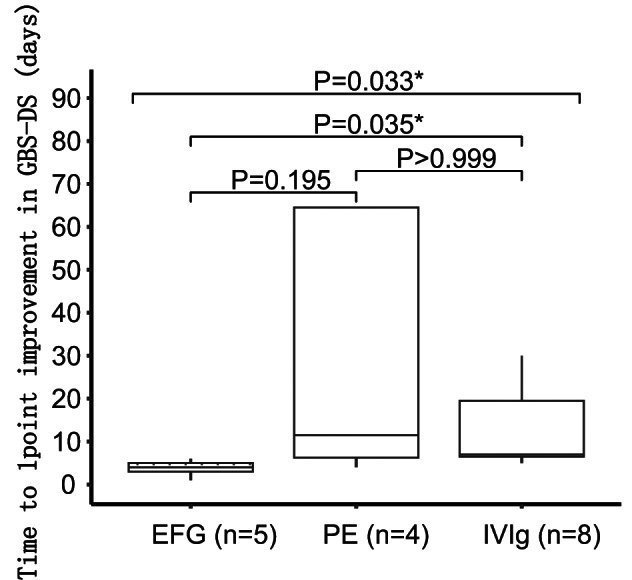
Pairwise comparison of time from immunotherapy initiation to one-point improvement in GBS-DS among IVIg, PE, and EFG groups( IVIg group: 7.00 [6.50, 19.50] days, PE group: 11.50 [6.25, 64.50] days and EFG group: 4.00 [3.00, 5.00] days, *p* = 0.033(Kruskal–Wallis test)), post-hoc pairwise comparisons with Bonferroni correction showed EFG group achieved a one-point improvement in GBS-DS significantly earlier than IVIg group (*p* = 0.035) and slightly higher than PE group(*p* = 0.195) *P < 0.05.

**Fig. 3:**
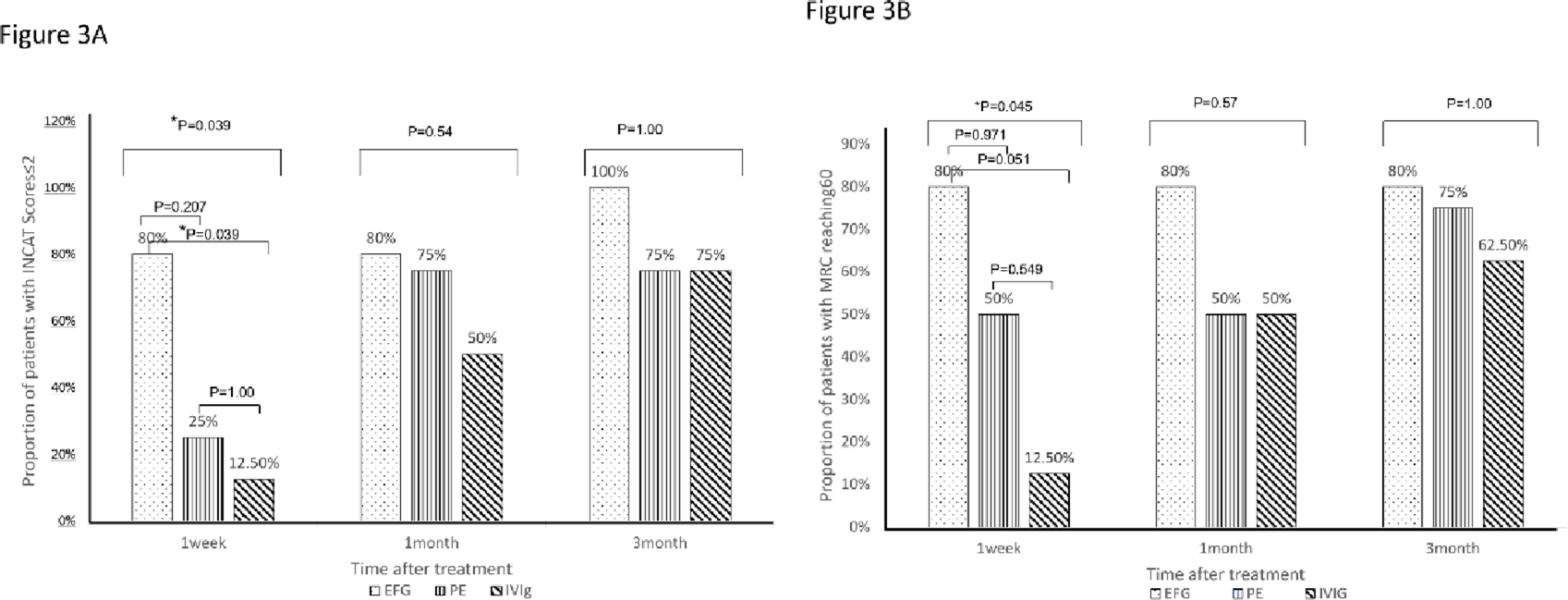
Pairwise comparison of the proportion of patients with INCAT scores ≤ 2 (**A**) and the proportion achieving full MRC scores (**B**) among IVIg, PE, and EFG groups at different follow-up times. Figure A: The proportion of patients with INCAT scores ≤ 2 showed significantly difference among three groups after1 week(*p* = 0.039, chi -square test), post-hoc pairwise comparisons with Bonferroni correction showed significantly difference between EFG group and IVIg group (*P* = 0.039).Figure B : the proportion achieving full MRC scores showed significantly difference anong three groups after 1 week(*p* = 0.045,chi-square test), post-hoc pairwise comparisons with Bonferroni correction showed a strong trend toward significance between EFG group and IVIg group (*P* = 0.051).

Further pairwise comparisons showed that EFG group achieved a one-point improvement in GBS-DS significantly earlier than IVIg group (*p* = 0.035) and slightly higher than PE group (Fig. 2). The proportion of patients with INCAT scores ≤ 2 was significantly higher in the EFG group compared with the IVIg group at the first week post-treatment (80% vs. 12.5%, *p* = 0.039), while the proportion of EFG group was higher than PE groups without statistically significant (80% vs. 25%, *p* = 0.207) (Fig. 3A). The proportion of patients in the EFG group achieving full MRC scores within one week was notably higher than in the IVIg group, showing a strong trend toward significance (80% vs. 12.5%, *p* = 0.051) (Fig. 3B).

**Fig. 4:**
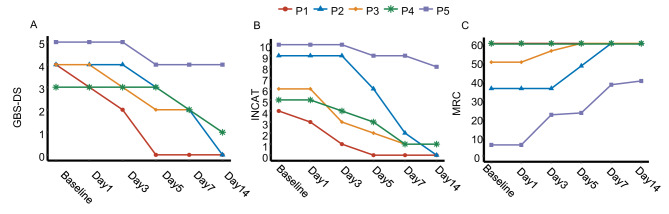
Individual longitudinal changes in GBS-DS (**A**), INCAT scores (**B**) and MRC scores (**C**) within 14 days after treatment in 5 patients of efgartigimod groups. P1, P4, and P5 received efgartigimod monotherapy, whereas patients P2 and P3 received PE followed by efgartigimod.

Among the 5 patients of EFG group: Patient 1 showed symptom improvement on day 1,recovered unaided walking on day 3, and reached an INCAT score ≤ 2 by day 3. Patient 2 showed symptom improvement on day 5, recovered unaided walking on day 7, and reached both an INCAT score ≤ 2 and full MRC score by day 7. Patient 3 showed symptom improvement on day 3, recovered unaided walking on day 5, and reached both an INCAT score ≤ 2 and full MRC score by day 5. Patient 4 showed symptom improvement on day 7, recovered unaided walking on day 7, and reached an INCAT score ≤ 2 by day 7. Patient 5 showed symptom improvement on day 5 but did not reach an INCAT score ≤ 2 or full MRC score within 14 days after treatment. (Fig. 4).

These results suggested that early and significant clinical improvement was observed in GBS patients who treated with efgartigimod.

### Atypical symptom resolution

Of the 2 patients with respiratory distress requiring intubation, 1 treated with efgartigimod had the tracheal tube removed on day 4 after treatment initiation, while 1 administered IVIg had the tube removed on day 5.

Among the 3 patients with pain, 2 treated with efgartigimod had symptom resolution on days 3 and 12 respectively, while the case administered PE had symptom resolution on day 3.

Of the 2 patients with facial palsy,1 treated with efgartigimod had symptom resolution on day 17 and the case administered IVIg had symptom resolution on day 90.

Among the 4 patients with dysphagia and dysarthria,1treated with efgartigimod had symptom resolution on day 3, while 2 administered with IVIg had symptom resolution on days 4 and 14 respectively and 1treated with PE had symptom resolution on day 60.

The patient with diplopia and ptosis experienced symptom resolution on day 18 following efgartigimod treatment.

Of the 2 patients with urinary retention,1 treated with PE had symptom resolution on day 7 and the case administered IVIg had symptom resolution on day 20.

Due to the limited number of patients in each group, statistical comparisons for symptom resolution were not performed.

### Safety assessment

During immunotherapy, 20% (1/5) of patients in the EFG group experienced adverse reactions, presenting as a mild skin rash. No adverse reactions were observed in the IVIg group. In the PE group, 50% (2/4) of patients experienced adverse reactions, both presenting as hypofibrinogenemia. No significant differences in adverse event rates were observed among the three groups.

## Discussion

Our study suggests that efgartigimod treatment for GBS offers a more rapid therapeutic effect compared to IVIg, as evidenced by the significantly shorter time to achieve a one-point improvement in the GBS-DS and significantly higher proportion of patients achieving favorable outcomes within 1-week post-treatment. Furthermore, the safety profile of EFG was comparable to that of traditional immunotherapies, with no increase in adverse reactions observed.

PE and IVIg have long been established as effective immunotherapies for GBS^23^. A multicenter controlled trial about PE in GBS showed that PE takes an average of 6 days to onset of motor recovery, takes an average of 18 days to begin weaning from ventilator support, and takes an average of 30 days to walk with assistance^8^. IVIg has also been shown to be at least as effective as PE in improving motor function, which is proven by a randomized trial^9^. The study reported that the median time to improvement by one grade was 27 days for IVIg and 41 days for PE^9^. In a special research about MFS, IVIg and PE seem not to have influenced MFS patients’ nature outcomes^27^.

In recent years, clinicians have also tried to treat GBS with novel monoclonal drugs, such as C5 complement against and FcRn-blocking agent^21,22,28,29^, especially in refractory GBS^30^. Eculizumab, a humanized monoclonal antibody against complement protein C5, inhibiting the formation of the membrane attack complex^31^, has shown potential benefits in treating GBS. In the JET-GBS trial, 61% of patients treated with eculizumab were able to walk independently by week 4, but no significant difference was reached compared to placebo group^29^.Efgartigimod, the noval FcRn-blocking agent, was proved to be effective in GBS treatment recently by several case reports^21,22,30,32,33^. A two-patient case report described rapid improvement in motor function within four weeks after two doses of efgartigimod monotherapy, which was the first report of GBS treatment by efgartigimod in 2024 ^21^.Subsequent reports have shown that in cases of refractory GBS or MFS where IVIg or PE treatment was ineffective, supplementing with 2–4 doses of efgartigimod, also can get clinical improvement from 3 to 14 days after efgartigimod and reach unaided walking within 2–4 weeks^21,30,32,33^.

Our study showed that the median time to improvement by one-point in GBS-DS score was 4 days for efgartigimod, ranging from 1 to 7 days. 3 patients with efgartigimod monotherapy took 1–5 days to show improvement. 2 patients who received PE followed by efgartigimod would take 5–7 days to show improvement. Our research and previous case reports have consistently shown that efgartigimod can indeed rapidly improve the symptoms of GBS patients, both in naive GBS patients and in refractory GBS cases.

Our study show that patients treated with efgartigimod exhibited a significantly earlier improvement in GBS-DS than that in the IVIg group while no statistically significant differences were observed between the Efgartigimod and PE. Additionally, the proportion of patients achieving an INCAT score ≤ 2 within one week was substantially higher in the Efgartigimod group (80%) than in the IVIg group (12.5%) and PE group (25%). The proportion of patients reaching full MRC scores within one week followed a similar trend. Our study suggests that efgartigimod may be able to halt the progression of the disease more quickly than IVIg to achieve symptomatic improvement. Unlike IVIg, which may take up to one or two weeks to exert its immunomodulatory effects, efgartigimod induces a faster decline in autoantibody levels, potentially leading to earlier clinical improvement. However, a 2025 observational study for naive GBS showed the Median time to improvement was.

4 days in the efgartigimod group, only slightly short than 8 days in the IVIg group. GBS-DS improve rates in week 1 of efgartigimod group was not higher than that of IVIg group^34^. Our study first compared the therapeutic indicators between efgartigimod and PE. It still showed that efgartigimod had earlier clinical improvement than PE, and the unaided walking rate after one week was also higher than that of PE. However, there was no significant difference. Whether efgartigimod can have a more rapid effect in treating GBS compared to IVIg and PE still needs to be confirmed by large-scale sample studies in the future. It is worth noting that this study did not find that efgartigimod was more effective than IVIg or PE in the subacute phase (at 1 month and 3 months), suggesting that the characteristic of efgartigimod is its faster onset, but the ultimate efficacy may be comparable to that of traditional treatments.

Beyond motor recovery, our study found efgartigimod facilitated the earlier resolution of atypical symptoms, including respiratory distress, pain, facial paralysis, dysphagia, and urinary retention. These results are highly consistent with the recent case report published, which mentioned that three GBS patients treated with efgartigimod experienced rapid improvement in atypical symptoms such as respiratory failure, diplopia, and dysphagia^32^. The rapid resolution of symptoms suggests efgartigimod has a broader immunomodulatory effect by reducing circulating IgG levels.

Efgartigimod is well-tolerated, with only one patient (20%) experiencing mild rash, compared to a higher incidence of hypofibrinogenemia (50%) in the PE group. No patients in the Efgartigimod group developed severe adverse reactions such as thrombosis, aseptic meningitis, or renal dysfunction, which are known risks associated with IVIg and PE. This is consistent with the previous studies ^18,34–36^.

Beyond efgartigimod, other FcRn-blocking agents and IgG-depleting strategies are under clinical development or already in use for autoimmune diseases, and may also have potential relevance for the treatment of GBS.

Although our results provide clinical evidence for the treatment of GBS with efgartigimod, there are still some limitations. our study was a retrospective study and limited by a relatively small cohort of 17 patients. Larger randomized controlled trials (RCTs) are needed to confirm our findings. In addition, our study did not conduct a classification and observation of the therapeutic effects based on the subtypes of GBS, different severity levels, as well as whether the GBS was naive or was refractory. We primarily focused on short-term outcomes (3-month follow-up). Future studies should evaluate long-term efficacy, particularly in preventing relapses or residual neurological deficits.

## Conclusion

This study provides preliminary evidence that efgartigimod offers early clinical improvement, extensive symptom relief, and a favorable safety profile in the treatment of GBS. Given these advantages, efgartigimod may serve as a valuable supplement to traditional therapies. Although the sample size was limited, the findings strongly support its potential as a promising new treatment option for GBS. However, larger-scale clinical trials are needed to further validate these results, establish optimal dosing regimens, and assess its long-term efficacy in preventing GBS-related disability.

## Supplementary Information

Below is the link to the electronic supplementary material.

Supplementary Material 1

## Data Availability

The datasets generated and/or analyzed during the current study are available from the corresponding author on reasonable request.

## References

[1] Illa, I. et al. Acute axonal Guillain-Barre syndrome with IgG antibodies against motor axons following parenteral gangliosides. *Ann. Neurol.* **38**, 218–224. 10.1002/ana.410380214 (1995). 7654069 10.1002/ana.410380214

[2] Wachira, V. K., Peixoto, H. M. & de Oliveira, M. R. F. Systematic review of factors associated with the development of Guillain-Barre syndrome 2007–2017: what has changed? *Trop. Med. Int. Health*. **24**, 132–142. 10.1111/tmi.13181 (2019). 30444562 10.1111/tmi.13181

[3] Matsui, N. et al. Guillain-Barre syndrome in a local area in Japan, 2006–2015: an epidemiological and clinical study of 108 patients. *Eur. J. Neurol.* **25**, 718–724. 10.1111/ene.13569 (2018). 29337417 10.1111/ene.13569

[4] Islam, Z. et al. High incidence of Guillain-Barre syndrome in children, Bangladesh. *Emerg. Infect. Dis.* **17**, 1317–1318. 10.3201/eid1707.102031 (2011). 21762603 10.3201/eid1707.101999 PMC3381380

[5] Shahrizaila, N., Lehmann, H. C. & Kuwabara, S. Guillain-Barre syndrome. *Lancet* **397**, 1214–1228. 10.1016/S0140-6736(21)00517-1 (2021). 33647239 10.1016/S0140-6736(21)00517-1

[6] Hughes, R. A., Swan, A. V. & van Doorn, P. A. Intravenous immunoglobulin for Guillain-Barre syndrome. *Cochrane Database Syst Rev* **2014**, CD002063 (2014). 10.1002/14651858.CD002063.pub6 10.1002/14651858.CD002063.pub2 14973982

[7] Schroder, A., Linker, R. A. & Gold, R. Plasmapheresis for neurological disorders. *Expert Rev. Neurother.* **9**, 1331–1339. 10.1586/ern.09.81 (2009). 19769448 10.1586/ern.09.81

[8] Efficiency of plasma. exchange in Guillain-Barre syndrome: role of replacement fluids. French Cooperative Group on Plasma Exchange in Guillain-Barre syndrome. *Ann. Neurol.* **22**, 753–761. 10.1002/ana.410220612 (1987). 2893583 10.1002/ana.410220612

[9] van der Meche, F. G. & Schmitz, P. I. A randomized trial comparing intravenous immune globulin and plasma exchange in Guillain-Barre syndrome. Dutch Guillain-Barre Study Group. *N Engl. J. Med.* **326**, 1123–1129. 10.1056/NEJM199204233261705 (1992). 1552913 10.1056/NEJM199204233261705

[10] Irani, D. N., Cornblath, D. R., Chaudhry, V., Borel, C. & Hanley, D. F. Relapse in Guillain-Barre syndrome after treatment with human immune globulin. *Neurology* **43**, 872–875. 10.1212/wnl.43.5.872 (1993). 8492939 10.1212/wnl.43.5.872

[11] Castro, L. H. & Ropper, A. H. Human immune globulin infusion in Guillain-Barre syndrome: worsening during and after treatment. *Neurology* **43**, 1034–1036. 10.1212/wnl.43.5.1034 (1993). 8492921 10.1212/wnl.43.5.1034

[12] Latov, N. Practice parameter: immunotherapy for Guillain-Barre syndrome: report of the Quality Standards Subcommittee of the American Academy of Neurology. *Neurology* **62**, 1653–1654. 10.1212/wnl.62.9.1653-a (2004). author reply 1654. 15136711 10.1212/wnl.62.9.1653-a

[13] Chevret, S., Hughes, R. A. & Annane, D. Plasma exchange for Guillain-Barre syndrome. *Cochrane Database Syst Rev* **2**, CD001798 (2017). 10.1002/14651858.CD001798.pub3 10.1002/14651858.CD001798.pub3 PMC6464100 28241090

[14] Dalakas, M. C. Intravenous immunoglobulin in autoimmune neuromuscular diseases. *JAMA* **291**, 2367–2375. 10.1001/jama.291.19.2367 (2004). 15150209 10.1001/jama.291.19.2367

[15] Heo, Y. A. & Efgartigimod *First Approval Drugs* **82**, 341–348 10.1007/s40265-022-01678-3 (2022). 35179720 10.1007/s40265-022-01678-3 PMC8855644

[16] Roopenian, D. C. & Akilesh, S. FcRn: the neonatal Fc receptor comes of age. *Nat. Rev. Immunol.* **7**, 715–725. 10.1038/nri2155 (2007). 17703228 10.1038/nri2155

[17] Howard, J. F. Jr. et al. Safety, efficacy, and tolerability of efgartigimod in patients with generalised myasthenia gravis (ADAPT): a multicentre, randomised, placebo-controlled, phase 3 trial. *Lancet Neurol.* **20**, 526–536. 10.1016/S1474-4422(21)00159-9 (2021). 34146511 10.1016/S1474-4422(21)00159-9

[18] Goebeler, M. et al. Treatment of pemphigus vulgaris and foliaceus with efgartigimod, a neonatal Fc receptor inhibitor: a phase II multicentre, open-label feasibility trial. *Br. J. Dermatol.* **186**, 429–439. 10.1111/bjd.20782 (2022). 34608631 10.1111/bjd.20782

[19] Broome, C. M. et al. Efficacy and safety of the neonatal Fc receptor inhibitor efgartigimod in adults with primary immune thrombocytopenia (ADVANCE IV): a multicentre, randomised, placebo-controlled, phase 3 trial. *Lancet* **402**, 1648–1659. 10.1016/S0140-6736(23)01460-5 (2023). 37778358 10.1016/S0140-6736(23)01460-5

[20] Allen, J. A. et al. Safety, tolerability, and efficacy of subcutaneous efgartigimod in patients with chronic inflammatory demyelinating polyradiculoneuropathy (ADHERE): a multicentre, randomised-withdrawal, double-blind, placebo-controlled, phase 2 trial. *Lancet Neurol.* **23**, 1013–1024. 10.1016/S1474-4422(24)00309-0 (2024). 39304241 10.1016/S1474-4422(24)00309-0

[21] Zhang, H. et al. Efgartigimod in the treatment of Guillain-Barre syndrome. *J. Neurol.* **271**, 3506–3511. 10.1007/s00415-024-12321-4 (2024). 38532142 10.1007/s00415-024-12321-4

[22] Zhou, J., Yu, W., Ding, S., Shi, C. & Liang, H. Resolution of acute motor axonal neuropathy in a patient after treatment with efgartigimod: A case report. *Med. (Baltim).* **103**, e40700. 10.1097/MD.0000000000040700 (2024). 10.1097/MD.0000000000040700 PMC11630930 39654182

[23] van Doorn, P. A. et al. European Academy of Neurology/Peripheral Nerve Society Guideline on diagnosis and treatment of Guillain-Barre syndrome. *Eur. J. Neurol.* **30**, 3646–3674. 10.1111/ene.16073 (2023). 37814552 10.1111/ene.16073

[24] Hughes, R. A. C., Cornblath, D. R., Jacobs, B. C. & van Doorn, P. A. Guillain-Barre Syndrome Disability Scale. *J. Peripher Nerv. Syst.* **30**, e70064. 10.1111/jns.70064 (2025). 41164964 10.1111/jns.70064 PMC12573220

[25] Breiner, A., Barnett, C. & Bril, V. INCAT disability score: a critical analysis of its measurement properties. *Muscle Nerve*. **50**, 164–169. 10.1002/mus.24207 (2014). 24723454 10.1002/mus.24207

[26] Kleyweg, R. P., van der Meche, F. G. & Schmitz, P. I. Interobserver agreement in the assessment of muscle strength and functional abilities in Guillain-Barre syndrome. *Muscle Nerve*. **14**, 1103–1109. 10.1002/mus.880141111 (1991). 1745285 10.1002/mus.880141111

[27] Mori, M., Kuwabara, S., Fukutake, T. & Hattori, T. Intravenous immunoglobulin therapy for Miller Fisher syndrome. *Neurology* **68**, 1144–1146. 10.1212/01.wnl.0000258673.31824.61 (2007). 17404197 10.1212/01.wnl.0000258673.31824.61

[28] Davidson, A. I. et al. Inhibition of complement in Guillain-Barre syndrome: the ICA-GBS study. *J. Peripher Nerv. Syst.* **22**, 4–12. 10.1111/jns.12194 (2017). 27801990 10.1111/jns.12194

[29] Misawa, S. et al. Safety and efficacy of eculizumab in Guillain-Barré syndrome: a multicentre, double-blind, randomised phase 2 trial. *Lancet Neurol.* **17**, 519–529. 10.1016/s1474-4422(18)30114-5 (2018). 29685815 10.1016/S1474-4422(18)30114-5

[30] Ripellino, P. et al. Efgartigimod as Add-On Treatment in Refractory Guillain-Barre Syndrome: A Case Report. *Eur. J. Neurol.* **32**, e70308. 10.1111/ene.70308 (2025). 40799081 10.1111/ene.70308 PMC12344377

[31] Thomas, T. C. et al. Inhibition of complement activity by humanized anti-C5 antibody and single-chain Fv. *Mol. Immunol.* **33**, 1389–1401. 10.1016/s0161-5890(96)00078-8 (1996). 9171898 10.1016/s0161-5890(96)00078-8

[32] Chen, S., Ou, R., Wei, Q., Zhao, B. & Chen, X. Sequential administration of efgartigimod shortened the course of Guillain-Barre syndrome: a case series. *Ther. Adv. Neurol. Disord*. **18**, 17562864251314746. 10.1177/17562864251314746 (2025). 40012687 10.1177/17562864251314746 PMC11863258

[33] Deng, M., Kong, Z., Wang, Y., Wang, X. & Li, T. Efgartigimod in the treatment of Guillain-Barre syndrome: case report. *Front. Immunol.* **16**, 1586663. 10.3389/fimmu.2025.1586663 (2025). 40977730 10.3389/fimmu.2025.1586663 PMC12443784

[34] Cai, H. et al. Intravenous Efgartigimod or Intravenous Immunoglobulin in Guillain-Barre Syndrome: An Observational Multicenter Study. *J. Peripher Nerv. Syst.* **30**, e70072. 10.1111/jns.70072 (2025). 41287201 10.1111/jns.70072

[35] Howard, J. F. Jr. et al. Safety, efficacy, and tolerability of efgartigimod in patients with generalised myasthenia gravis (ADAPT): a multicentre, randomised, placebo-controlled, phase 3 trial. *Lancet Neurol.* **20**, 526–536. 10.1016/s1474-4422(21)00159-9 (2021). 34146511 10.1016/S1474-4422(21)00159-9

[36] Newland, A. C. et al. Phase 2 study of efgartigimod, a novel FcRn antagonist, in adult patients with primary immune thrombocytopenia. *Am. J. Hematol.* **95**, 178–187. 10.1002/ajh.25680 (2020). 31821591 10.1002/ajh.25680 PMC7004056

