# Supplementary material for: Rapid neurological recovery in Guillain-Barré syndrome treated with efgartigimod
Source: Sci Rep. 2026 Mar 19;16:14128. doi: 10.1038/s41598-026-44163-7 (PMC13136387; doi:10.1038/s41598-026-44163-7)
Supplement: Supplementary file 1 — Supplementary Material 1 [file 41598_2026_44163_MOESM1_ESM.docx]

| Table S1 clinical imformations of 5 patients in EFG group | | | | | |
| --- | --- | --- | --- | --- | --- |
|  | Patient 1 | Patient 2 | Patient 3 | Patient 4 | Patient 5 |
| sex | M | F | F | M | M |
| age | 61 | 42 | 78 | 47 | 26 |
| GBS subtype | MFS/GBS | GBS | GBS | MFS | GBS |
| baseline GBSDS | 4 | 4 | 4 | 3 | 5 |
| baseline INCAT | 2 | 9 | 6 | 5 | 10 |
| baseline MRC | 60 | 36 | 50 | 60 | 6 |
| initial symptom | ataxia | pain | pain | dizziness，diplopia | limb weakness |
| core symptom | ataxia | limb weakness | limb weakness | ataxia | limb weakness |
| other symptom | dysphagia | facial paralysis | / | ptosis | uroschesis, dyspnea |
| albuminocytologic dissociation | No | Yes | Yes | Yes | No |
| antibody | GQ1b，GT1a | / | / | GQ1b、GT1a、GM2 | GM3 |
| treatment | efgartigimod | efgartigimod | efgartigimod | PE every other day for 3 times,followed by efgartigimod after six days | PE every other day for 7 times,followed by efgartigimod the next day |
| treatment days from onset | 8 | 4 | 4 | 16 | 27 |
| M: male; F :female; GBS:Guillain-Barre syndrome; MFS: Miller Fisher syndrome; GBS/MFS: GBS/MFS overlap; INCAT: Inflammatory Neuropathy Cause and Treatment; GBS-DS: GBS disability grade score; MRC: Medical Research Council scores; PE:plasma exchange; | | | | | |
